# Assessing Susceptibility from Early-Life Exposure to Carcinogens

**DOI:** 10.1289/ehp.7667

**Published:** 2005-04-07

**Authors:** Hugh A. Barton, V. James Cogliano, Lynn Flowers, Larry Valcovic, R. Woodrow Setzer, Tracey J. Woodruff

**Affiliations:** 1 Office of Research and Development, National Center for Computational Toxicology, U.S. Environmental Protection Agency, Research Triangle Park, North Carolina, USA; 2 Office of Research and Development, National Center for Environmental Assessment, U.S. Environmental Protection Agency, Washington, DC, USA; 3 Office of Policy, Economics, and Innovation, U.S. Environmental Protection Agency, San Francisco, California, USA

**Keywords:** cancer, children, early-life exposure, exposure, mode of action, risk assessment, susceptible populations

## Abstract

Cancer risk assessment methods currently assume that children and adults are equally susceptible to exposure to chemicals. We reviewed available scientific literature to determine whether this was scientifically supported. We identified more than 50 chemicals causing cancer after perinatal exposure. Human data are extremely limited, with radiation exposures showing increased early susceptibility at some tumor sites. Twenty-seven rodent studies for 18 chemicals had sufficient data after postnatal and adult exposures to quantitatively estimate potential increased susceptibility from early-life exposure, calculated as the ratio of juvenile to adult cancer potencies for three study types: acute dosing, repeated dosing, and lifetime dosing. Twelve of the chemicals act through a mutagenic mode of action. For these, the geometric mean ratio was 11 for lifetime exposures and 8.7 for repeat exposures, with a ratio of 10 for these studies combined. The geometric mean ratio for acute studies is 1.5, which was influenced by tissue-specific results [geometric mean ratios for kidney, leukemia, liver, lymph, mammary, nerve, reticular tissue, thymic lymphoma, and uterus/vagina > 1 (range, 1.6–8.1); forestomach, harderian gland, ovaries, and thyroid < 1 (range, 0.033–0.45)]. Chemicals causing cancer through other modes of action indicate some increased susceptibility from postnatal exposure (geometric mean ratio is 3.4 for lifetime exposure, 2.2 for repeat exposure). Early exposures to compounds with endocrine activity sometimes produce different tumors after exposures at different ages. These analyses suggest increased susceptibility to cancer from early-life exposure, particularly for chemicals acting through a mutagenic mode of action.

The cancer database used by the U.S. Environmental Protection Agency (EPA) and other agencies for risk assessment for exposure to carcinogens focuses on adults and adult exposures. Much cancer epidemiology comes from occupational studies and rodent cancer studies, which were designed to last approximately a lifetime (2 years) beginning after sexual maturity. Cancer risks from childhood exposures to chemicals are generally analyzed using methods based on exposure to adults, which assumes chemicals are equally potent for inducing risks of exposures in both early life and later life. Animal and human data suggest that further analysis is warranted to determine whether early-life exposure results in increased susceptibility to cancer compared with adult exposures (Anderson et al. 2000; National Research Council 1993). There is extensive literature demonstrating that exposures early in life (i.e., transplacental or *in utero*, early postnatal, and lactational) in animals can result in the development of cancer (reviewed in Anderson et al. 2000; Della Porta and Terracini 1969; Druckrey 1973; Rice 1979; Rice and Ward 1982; Toth 1968; Vesselinovitch 1983; Vesselinovitch et al. 1979a). However, except for data on radiation and prenatal exposure to diethylstilbesterol (DES), there are virtually no human data adequate for quantitative analysis.

Standard animal bioassays generally begin dosing after the animals are 6–8 weeks of age, when many organs and systems are relatively mature, although substantial growth in body size continues thereafter. Reviews comparing perinatal carcinogenesis bioassays with standard bioassays for a limited number of chemicals (McConnell 1992; U.S. EPA 1996) have concluded that the same tumor sites usually are observed after either perinatal or adult exposure, and that perinatal exposure in conjunction with adult exposure usually increases the incidence of tumors or reduces the latent period before tumors are observed.

There is limited evidence to inform the mode(s) of action leading to differences in tumor type and tumor incidence after early-life exposure compared with exposure later in life. Differences in the capacity to metabolize and clear chemicals at early ages can result in larger or smaller internal doses of the active agent(s), either increasing or decreasing risk (Ginsberg et al. 2002; Renwick 1998). Mechanistic data supporting early-life susceptibility to DNA damaging mutagenic chemicals include increased formation of DNA adducts after exposure to vinyl chloride (Laib et al. 1989; Morinello et al. 2002a, 2002b), increased induction of micronuclei in fetal tissues (Hayashi et al. 2000), and increased mutations in brains of transgenic mice exposed to ethylnitrosourea (ENU; Slikker et al. 2004). The neonatal mouse model for carcinogenesis, which uses two doses before weaning followed by observation of tumors at 1 year, shows carcinogenic responses for mutagenic carcinogens (Flammang et al. 1997; Fujii 1991; McClain et al. 2001).

Current understanding of biologic processes involved in carcinogenesis leads to a reasonable expectation that children are more susceptible to some carcinogenic agents than adults (Anderson et al. 2000; Birnbaum and Fenton 2003; Ginsberg 2003; Miller et al. 2002; Scheuplein et al. 2002). Several aspects potentially lead to increased childhood susceptibility. More frequent cell division during development can result in enhanced fixation of mutations because of the reduced time available for repair of DNA lesions, and clonal expansion of mutant cells gives a larger population of mutants (Slikker et al. 2004). Some components of the immune system are not fully functional during development (Holladay and Smialowicz 2000; Holsapple et al. 2003). Hormonal systems operate at different levels during various life stages (Anderson et al. 2000). Induction of developmental abnormalities may result in a predisposition to carcinogenic effects later in life (Birnbaum and Fenton 2003; Fenton and Davis 2002).

Finally, theoretical analyses suggest that differential susceptibility would depend, in part, on the mode of action [i.e., at what step in the cancer process(es) the chemical was acting] and that the lifetime average daily dose may underestimate or overestimate the cancer risk when exposures are time dependent (Goddard and Krewski 1995; Murdoch et al. 1992).

We reviewed the available scientific literature to determine the extent of potential increased susceptibility from early-life exposure. We evaluated potential susceptibility by individual study and tumor type rather than a combined analysis of all data by certain categories (e.g., gender) (Hattis et al. 2004). Further supporting evidence from ionizing radiation is given in the Appendix (http://ehp.niehs.nih.gov/docs/2005/7667/app.pdf). Although there is evidence showing that prenatal exposures can result in tumors later in life (Anderson et al. 2000; Birnbaum and Fenton 2003; Diwan et al. 1992; Hatch et al. 1998; Waalkes et al. 2003), this analysis focuses only on exposures in animals occurring postnatally up to approximately 5–8 weeks of age.

## Materials and Methods

### Procedures

#### Data sources for animal studies.

We identified initial studies for consideration through review articles and a search of the National Toxicology Program database (NTP 2003). A literature search was conducted using key words and MeSH headings from the PubMed database (PubMed 2004) from studies identified in the available reviews. The chemicals considered and then included for quantitative evaluation are listed in Table 1 and in Supplementary Table S1 (http://ehp.niehs.nih.gov/docs/2005/7667/sup.pdf).

We reviewed abstracts or papers to determine if a study provided information that could be used for quantitative analysis based on the following criteria:

- Exposure groups at different postnatal ages in the same study or same laboratory, if not concurrent (to control a large number of potential cross-laboratory experimental variables including pathologic examinations)
- Same strain/species (to eliminate strain-specific responses confounding age-dependent responses)
- Approximately the same dose within the limits of diets, and drinking water intakes that obviously can vary with age (to eliminate dose-dependent responses confounding age-dependent responses).
- Similar or identical period for tumor expression after exposures at different ages—variations of around 10–20% in time to sacrifice are acceptable arising from sacrifice at > 1 year for all groups exposed at different ages, where early-life exposure can occur up to about 7 weeks (to control for confounding differential periods for tumor expression with age-dependent changes in tumor incidences).
- Postnatal exposure for juvenile rats and mice at ages younger than the standard 6–8 week start for bioassays; studies that have postnatal exposure were included even if they also involved prenatal exposure, but studies with only prenatal (in utero) exposures are not part of the present analysis.
- “Adult” rats and mice exposure beginning at approximately 6–8 weeks of age, that is, comparable with the age at initiation of a standard cancer bioassay. Studies in other species were used as supporting evidence, because they are relatively rare and the determination of the appropriate comparison ages across species is not simple.
- Number of affected animals and total number of animals examined must be available or reasonably reconstructed for control, young, and adult groups (i.e., studies reporting only percent response or not including a control group would be excluded unless a reasonable estimate of historical background for the strain was obtainable).

Supplementary Tables S2 and S3 (http://ehp.niehs.nih.gov/docs/2005/7667/supp.pdf) include information used for the calculations. Pertinent information on species, sex, dosing regimen, and tumor incidence is given.

**Figure 1:**
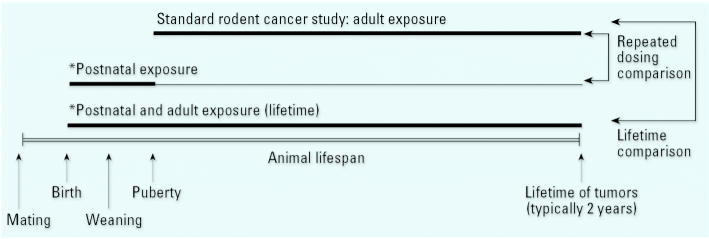
Schematic representation of several cancer study designs reported in the evaluated literature. The standard rodent bioassay begins after puberty, and exposures continue for about 2 years. Repeated-dosing studies typically dose during the postnatal period, with observations for tumors at approximately 2 years. Lifetime studies combine postnatal and adult exposures, sometimes beginning with *in utero* exposure. Acute studies (not shown) generally involve one or a few exposure during the *in utero*, preweaning, prepubertal, and adult periods. The adult tumors were often evaluated much earlier than 2 years *Can also include prenatal exposure.

The literature includes studies that can be divided roughly into three types of exposure scenarios (Figure 1): repeated exposures for the early postnatal to juvenile period compared with chronic later-life dosing; lifetime (i.e., combined perinatal and adult) exposure compared with chronic later-life dosing; and acute exposures such as a single intraperitoneal or subcutaneous injection for both early-life and later-life dosing.

#### Evaluating the mode of action of carcinogens.

Chemicals were classified into categories based on evaluating the mode of action using a weight-of-evidence approach. Determination of carcinogens that are operating by a mutagenic mode of action entails evaluation of short-term testing results for genetic end points, metabolic profiles, physicochemical properties, and structure–activity relationship (SAR) analyses in a weight-of-evidence approach (Dearfield et al. 1991; U.S. EPA 1986 U.S. EPA 1991; Waters et al. 1999), as has been done for several chemicals (e.g., Dearfield et al. 1999; McCarroll et al. 2002; U.S. EPA 2000). Key data for a mutagenic mode of action may be evidence that the carcinogen or a metabolite is DNA reactive and/or has the ability to bind to DNA. Also, mutagenic carcinogens usually produce positive effects in multiple test systems for different genetic end points, particularly gene mutations, and in tests performed *in vivo* that generally are supported by positive tests *in vitro*. Additionally, carcinogens may be identified as operating via a mutagenic mode of action if they have properties and SARs similar to those of established mutagenic carcinogens. Those with a mutagenic mode of action are identified in Table 1.

### Quantitative Methods

To estimate the potential difference in susceptibility between early-life and adult exposure, we calculated the ratio of the estimated cancer potency from early-life exposure compared with the estimated cancer potency from adult exposure. The cancer potency was estimated from a one-hit model, or a restricted form of the Weibull model, which is commonly used to estimate cumulative incidence for tumor onset. The general form of the equation is as follows:


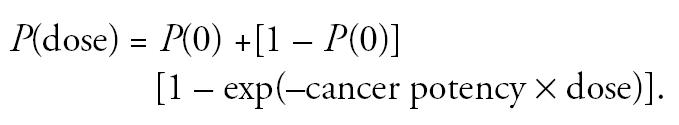


Juvenile and adult cancer potencies and the ratio of the two were calculated by fitting this model to the data for each age group. The model fit depended upon the design of the experiment that generated the data. Two designs need to be handled separately: repeat and acute exposure and lifetime exposures.

For the first case, the model equations are as follows:


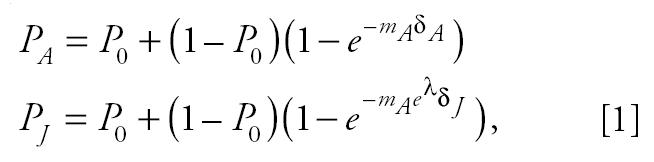


where subscripts A and J refer to the adult and juvenile period, respectively; λ is the natural logarithm of the juvenile:adult cancer potency ratio; *P*_0_ is the fraction of control animals with the particular tumor type being modeled; *P*_x_ is the fraction of animals exposed in age period *x* with the tumor; *m*_A_ is the cancer potency, the rate of accumulation of “hits” per unit of time for adults; and δ_x_ is the duration or number of exposures during age period x. For a substantial number of data sets (acute exposures), δ_J_ = δ_A_ = 1. We are interested in determining λ , which is the logarithm of the estimated ratio of juvenile to adult cancer potencies, a measure of potential susceptibility for early-life exposure.

For the lifetime exposures, the model equations need to take into account that exposures were initiated in the juvenile period continue through the adult period. The model equations for the fraction of animals exposed only as adults with tumors in this design is the same as in the first design, but the fraction of animals whose first exposure occurred in the juvenile period is


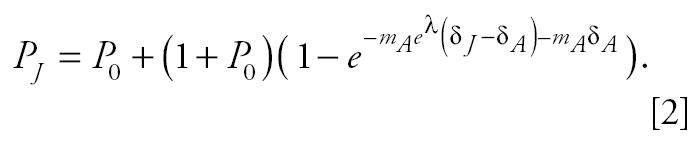


All symbols in Equation 2 have the same interpretation as their counterparts in Equation 1, but now δ_J_ includes the duration of exposure during the juvenile period as well as the subsequent adult period.

Parameters m_A_ and λ in these models were estimated using Bayesian methods ( e.g., see Carlin and Louis 2000), and all inferences about the ratios were based on the marginal posterior distribution of λ. The data for estimating each ratio were in the form of numbers of animals tested and number affected for each of control, juvenile-exposed, and adult-exposed animals, and duration of exposure for each of the juvenile-exposed and adult-exposed groups. A few data sets had separate control groups for the juvenile-exposed and adult-exposed groups, and Equations 1 and 2 were modified accordingly. The likelihood for the parameters m_A_ and λ in the model was the product of three (or four, if there were two control groups) binomial probabilities: the number of animals with tumors in the adult control group, in the juvenile control group, in the juvenile-exposed group, and in the adult-exposed group. The prior for P_0_ (the fraction of control animals with a particular tumor) was right triangular, which assumes incidences should be relatively low. The effect of exposure in adults is quantified by the extra risk, Q, where the probability that an animal has a tumor is P_0_ + (1 – P_0_)Q. So, from Equation 1, Q was given a uniform prior on the interval (0,1), reflecting total ignorance about the extra risk of adult exposure. Finally, the prior for λ was Gaussian with mean 0 (corresponding to a median or geometric mean ratio of 1) and standard deviation of 3. The prior for the log ratio has some influence over the posterior estimates for the ratio of juvenile to adult potency. The magnitude of that influence depends on the amount of support in the data for different values of the log ratio. This potential influence is further discussed with illustrations in the Appendix (http://ehp.niehs.nih.gov/docs/2005/7667/app.pdf).

To examine the sensitivity of the estimates to choice of the prior, values were re-estimated using a prior with a larger standard deviation. This was initially chosen to be 9, but for some of the lifetime exposure and acute exposure studies, a standard deviation of 9 led to numerical problems integrating the posterior, so a prior with a standard deviation of 6 was used in these cases. Sensitivity to the choice of prior was evaluated by looking at changes of individual log ratios and their variances and through differences in geometric means.

The posterior distribution for the unknown parameters in these models is the product of the likelihood from the data and the priors (the “unnormalized” prior), divided by a normalization constant that is the integral of the unnormalized prior over the ranges of all the parameters. This normalization constant was computed using numerical integration, as were posterior means and variances and marginal posterior quantiles for the log ratio λ. All numerical computations were carried out in the R statistical programming language (version 1.8.1; R Development Core Team 2003).

This method produced a posterior mean of ratio of the early-life to adult cancer potency, which is an estimate of the potential susceptibility of early-life exposure to carcinogens. Ratios > 1 indicated greater susceptibility from early-life exposure. Ratios < 1 indicated less susceptibility from early-life exposure. Summaries of the individual ratios from each of the dose groups from the different experiments for different groupings were also calculated (e.g., for all acute mutagenic chemicals). The summary ratios were constructed from the individual ratios within a group by inverse variance-weighting the means of each ratio. The individual means were weighted by using reciprocals of posterior variances, so ratios with more variance were given less weight in the summary ratios. Exponentiating the resulting variance-weighted mean yielded variance-weighted geometric means of ratios.

## Results

A review of the literature identified several hundred references reporting more than 50 chemicals able to cause cancer after perinatal exposure in animals [Supplementary Table S1 (http://ehp.niehs.nih.gov/docs/2005/7667/supp.pdf)]. Often, these studies demonstrated carcinogenesis after perinatal exposure but did not directly compare exposures in adults. A large number of studies address in utero exposures only. Studies across laboratories often varied in their use of animal strains [e.g., for 3′-azido-3′-deoxythymidine (AZT) studies, Diwan et al. (1999) used CD-1 mice, whereas the National Toxicology Program (NTP 1999) used B6C3F_1_ mice] or had different periods of tumor follow-up (e.g., tamoxifen and uterine tumors in Carthew et al. 2000). Because of these factors, many of these chemicals in Supplementary Table S1 (http://ehp.niehs.nih.gov/docs/2005/7667/supp.pdf) were not evaluated quantitatively. Studies assessing radiation in animals exist, but lack of uniformity regarding radiation doses, gestational age at exposure, and the animal strains used make it difficult to make comparisons across studies (Preston et al. 2000).

Some of the studies that did not have sufficient information for quantitative evaluation provide important supporting information for early-life susceptibility. Increased multiplicity of colon tumors was observed after earlier versus later azoxymethane exposures (Paulsen et al. 2003). Shortened mammary tumor latency after estradiol exposure occurred for exposures between 8 and 18 weeks as opposed to exposures earlier or later in life (Yang et al. 2003), consistent with results for dimethylbenz[*a*]anthracene (DMBA; Meranze et al. 1969). Notable examples exist of developmental windows leading to cancer susceptibilities that were not observable in adults. Several potent estrogens, including DES, tamoxifen, and genistein, produce uterine tumors with early postnatal exposures of mice, although there also appear to be strain-dependent differences in the tumor sites in adult mice (Gass et al. 1964; Greenman et al. 1990; Newbold et al. 1990, 1997, 2001). Developmental susceptibilities are believed to play a key role in effects observed with saccharin (Cohen et al. 1995; Whysner and Williams 1996) and ascorbate (Cohen et al. 1998; NTP 1983), with bladder tumors arising only when early-life exposures occurred; studies with other food additives did not find cancers after either adult-only or combined early-life and adult exposures (U.S. EPA 1996). Finally, central nervous systems tumors appear highly dependent upon exposures to ENU and several other chemicals during appropriate developmental windows, particularly prenatally, as observed in several species including rat, mouse, and opossum (Jurgelski et al. 1979; Rice 1979; Rice and Ward 1982).

### Quantitative Evaluation of the Database

Studies (or groups of studies from a single laboratory on a given chemical) that provided quantitative data were identified for 18 chemicals [Table 1; Supplementary Tables S2, S3 (http://ehp.niehs.nih.gov/docs/2005/7667/supp.pdf)]. Nine chemicals involved repeated exposures during early postnatal and adult life stages, eight chemicals had lifetime exposure starting in the juvenile period and adult-only exposure, and eight chemicals used acute exposures (typically single doses) at different ages (Table 1). Some studies evaluated single tissues for tumors (e.g., only liver), whereas others evaluated multiple tissues. Mice, rats, or both species and sometimes multiple strains were tested.

### Carcinogens with a Mutagenic Mode of Action

The most informative database on early–life-stage susceptibility exists for chemicals with a well accepted mutagenic mode of action and includes both acute-exposure and repeated-exposure studies involving periods of perinatal and/or chronic exposure.

#### Repeat and lifetime exposure studies of mutagenic chemicals.

Studies comparing repeated dosing for early postnatal, juvenile, adult time periods, or lifetime exposures exist for six mutagens [benzidine, diethylnitrosamine (DEN), 3-methylcholanthrene, safrole, urethane, and vinyl chloride; Tables 1, 2, 3; Supplementary Table S4 (http://ehp.niehs.nih.gov/docs/2005/7667/supp.pdf)]. DEN and urethane also had acute-dosing studies. These chemicals all require metabolic activation to the active carcinogenic form.

**Figure 2:**
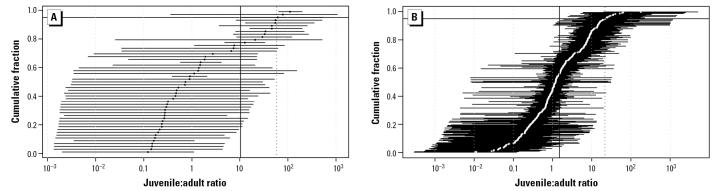
Posterior geometric means and 95% confidence intervals for the ratios of juvenile to adult cancer potency for carcinogens acting primarily through a mutagenic mode of action. (A) Repeated and lifetime exposure studies (geometric mean in black). (B) Acute exposure studies mutagens (geometric mean in white). The horizontal lines to the left and right of each geometric mean correspond to 95% confidence limits. The vertical solid line represents the geometric mean; the horizontal solid line represents the 95th percentile; the vertical dotted line is the geometric mean of the 95th percentile. The geometric mean for repeat and lifetime exposures is 10.4; for acute exposures the geometric mean value is 1.5.

For the repeated-dosing studies, the ratios of juvenile to adult cancer potency ranged from 0.12 to 111 with a geometric mean ratio of 11 (Tables 1, 2, 4; Figure 2). For the lifetime studies, the ratios ranged from 0.18 to 79 with a geometric mean of 8.7 [Tables 3, 4; Supplementary Table S5 (http://ehp.niehs.nih.gov/docs/2005/7667/supp.pdf); Figure 2]. The geometric mean combining the repeated and lifetime data for six chemicals was 10 (Table 4). Calculations based upon even broader, less informative prior distributions (e.g., SD = 6 or 9) gave higher estimates for the geometric means, 17 and 20, for repeated and lifetime studies, respectively, and wider ranges (0.0011–115.2 for repeated-dosing studies, 0.0014–157.78 for lifetime studies). The prior estimates appear to have a greater influence on the estimates of λ (natural log of the ratio of juvenile to adult cancer potency) from the lifetime study designs, reflecting the relative insensitivity to this parameter, as described further in the Appendix (http://ehp.niehs.nih.gov/docs/2005/7667/app.pdf). For benzidine and safrole, there was a notable sex difference, with high liver tumor incidence observed for early postnatal exposures of male, but not female, mice.

#### Acute-dosing studies of mutagenic chemicals.

Acute-dosing studies are available for eight mutagenic chemicals that were administered to mice or rats (benzo[*a*]pyrene, dibenzanthracene, DEN, DMBA, dimethylnitrosamine, ENU, N-methylnitrosourea [MNU], and urethane; Table 1). Except for ENU and MNU, these compounds require metabolic activation to their active carcinogenic forms.

Early acute exposures often resulted in higher potency, with increased early susceptibilities up to 178-fold (ratios of juvenile to adult potencies range from 0.011 to 178; geometric mean, 1.5) [Figure 2; Table 4; Supplementary Table S6 (http://ehp.niehs.nih.gov/docs/2005/7667/supp.pdf)]. Use of a broader prior distribution for λ (natural log of the ratio of juvenile to adult cancer potency) had no effect on the overall geometric mean (1.5) because the data highly informed the posterior distributions, although the range of individual ratios changed (0.00008–2,055). In studies comparing exposures on specific postnatal days 1, 15, and 42, general age-dependent declines in susceptibility of tumor response were observed, for example, benzo[*a*]pyrene (liver tumors), DEN (liver tumors), ENU (liver and nervous system tumors), and urethane (liver and lung tumors). Although generally the ratios for day 1 and day 15 time points were higher than those for later time points, in several cases similar tumor incidence was observed at both of these early times [e.g., ENU-induced kidney tumors; Supplementary Table S6 (http://ehp.niehs.nih.gov/docs/2005/7667/supp.pdf)].

Although the degree of susceptibility generally declines with age, there are exceptions, such as for pubertal periods of tissue development. Meranze et al. (1969) reported 8% mammary tumors after a single dose of DMBA at < 2 weeks of age, 56% if given once to animals between 5 and 8 weeks of age, and 15% when given once to 26-week-old rats. Thus, a ratio of 7.1 is obtained when comparing susceptibilities of 5- to 8-week-old and 26-week-old rats compared with a ratio of 0.2 when comparing the exposure at 2 weeks versus 26 weeks [Table 4; Supplementary Table S6 (http://ehp.niehs.nih.gov/docs/2005/7667/supp.pdf)]. A similar effect was observed by Russo et al. (1979; Supplementary Table S3 (http://ehp.niehs.nih.gov/docs/2005/7667/supp.pdf). This observation corresponds well with pubertal development of the mammary tissue, with ovarian function commencing between 3 and 4 weeks [after the < 2-week time point in the Meranze et al. (1969) study], and mammary ductal growth and branching occurring such that it is approximately two-thirds complete by week 5, consistent with the 5- to 8-week sensitive period of Meranze et al. (Silberstein 2001).

Early-life susceptibility of different tissues varies substantially in the acute studies (Table 4). It should be noted that the target tissues and tissues evaluated vary with chemical, so the number of chemicals for which data are available varies for each tissue. Several tissues have geometric mean ratios > 1, including kidney, leukemia, liver, lymph, mammary, nerve, reticular tissue, thymic lymphoma, and uterus/vagina. Some of these, such as the nerve and mammary tumors, appear to have a very specific window of susceptibility, and the ratios were much higher if the exposure occurred during this window. Tissues with mean ratios < 1 include forestomach, harderian gland, ovaries, and thyroid. Lung has a geometric mean of 1. Many of the studies produced very high lung tumor responses regardless of age, so the results are difficult to interpret, as illustrated by the dose–response data with urethane in Rogers (1951), in which the increased early susceptibility is only apparent when the dose is low. The large numbers of studies with high lung tumor responses at all ages are a major contributor to the differences in the geometric means for the acute and repeated-dosing studies.

#### Carcinogens with modes of action other than mutagenicity.

Studies comparing tumors observed at the same sites after early postnatal and chronic adult exposures in a single protocol were available for six chemicals that do not act through a mutagenic mode of action [amitrole, dichlorodiphenyltrichloroethane (DDT), dieldrin, ethylene thiourea (ETU), diphenylhydantoin (DPH), polybrominated biphenyls (PBB); Table 5]. These chemicals cause tumors through several different, not necessarily well-defined, modes of action. For example, thyroid hormone disruption by ETU causes thyroid tumors; some PBBs act through the aryl hydrocarbon receptor, whereas other PBBs are phenobarbital-like pleiotrophic inducers of liver enzymes and liver tumors. Three of these studies evaluated only mouse liver tumors (amitrole, DDT, dieldrin), whereas the other three evaluated a large number of tissues in both mice and rats (ETU, DPH, PBB). No acute-dosing studies were identified for these agents; such protocols are generally considered largely nonresponsive for modes of action other than mutagenicity and potent estrogenicity (e.g., DES).

## Discussion

The database overall is of modest size (particularly compared with the number of chemicals that have been studied in adult occupational epidemiologic studies or chronic bioassays). Information on different life-stage susceptibilities to cancer risks for humans exists for ionizing radiation [Appendix (http://ehp.niehs.nih.gov/docs/2005/7667/app.pdf)]. The effects on cancer induction by chemical mutagens at different life stages are derived from laboratory animal studies. Although the induction of cancer by ionizing radiation and chemical mutagens are not identical processes, both involve direct damage to DNA as critical causal steps in the process. In both cases, the impacts of early exposure can be greater than the impacts of later exposures. As indicated in Table 5 and Supplementary Tables S7–S10 (http://ehp.niehs.nih.gov/docs/2005/7667/supp.pdf), A-bomb survivors exhibit different life-stage dependencies at different tumor sites, although there are apparent differences at some sites. However, it is clear that the total radiation-related tumor incidence showed a general slow decline with age at exposure. The mutagenic chemical studies in rodents similarly support a general decline in cancer risk with age of exposure and similarly show differences for individual tumor sites. In general, the first 2 or 3 postnatal weeks in mice and rats appeared to be the most sensitive.

Analyses of the difference in cancer risk from exposures during different lifetime periods ideally need to address both the period of potential susceptibility and the magnitude of the susceptibility. Available studies used a variety of different study designs [Supplementary Tables S2, S3 (http://ehp.niehs.nih.gov/docs/2005/7667/supp.pdf)], which can be valuable because they provide different information. However, variations in study design can result in a lack of comparability across chemicals and can limit information on the consistency of effects with different chemicals acting through different modes of action. The acute-dosing (largely single-dose) studies are valuable because they involve identical exposures with explicitly defined doses and time periods demonstrating that differential tumor incidences arise exclusively from age-dependent susceptibility.

The repeated-dosing studies with exposures during early postnatal or adult lifetime provide useful information on the relative impact of repeated exposures at different life stages and may be more likely to have exposure occur during a window of susceptibility, if there is one. One notable difference in study designs was that studies with repeated early postnatal exposures were included in the analysis even if they also involved earlier maternal and/or prenatal exposure, whereas studies addressing only prenatal exposure were not otherwise a part of this analysis. The impact of this is limited because it applies to the lifetime safrole study and the studies with PBB, DPH, and ETU. Another notable difference among studies involved the tissues that were evaluated for tumors: some studies focused on a single tissue, particularly liver, whereas others evaluated multiple tissues.

This analysis assumes that the doses animals received during the different periods of repeated dose studies were similar. This assumption is a limitation because these studies involved exposures via lactation, drinking water, diet, or inhalation, which potentially deliver different doses at different life stages. However, the range of the magnitudes of the tumor incidence ratios of juvenile to adult exposures is similar for the repeated-dosing studies for chemicals with a mutagenic mode of action [0.12–111; geometric mean, 11; Tables 2, 4; Supplementary Table S5 (http://ehp.niehs.nih.gov/docs/2005/7667/supp.pdf), lifetime-dosing studies [0.28–79; geometric mean, 8.7; Tables 3, 4; Supplementary Table S5 (http://ehp.niehs.nih.gov/docs/2005/7667/supp.pdf)], and acute-dosing studies [0.01–178; geometric mean, 1.5; Table 4; Supplementary Tables S5, S6 (http://ehp.niehs.nih.gov/docs/2005/7667/supp.pdf)], suggesting that differences in dosing are not the sole determinant of the increased incidence of early tumors.

Because these comparisons include chemicals with different tissue specificities, it is useful to consider the liver as a target organ affected by all these chemicals; in so doing, even greater consistency is observed. The range of the magnitudes of the liver tumor potency ratios of juvenile to adult exposures of mutagenic chemicals is similar for the repeated-dosing studies (geometric mean, 41.8; range, 0.12–111; Table 2), lifetime-dosing studies (geometric mean, 14.9; range, 0.47–79; Table 3), and acute-dosing studies (geometric mean, 8.1; range, 0.1–40; Table 4). In some cases, windows of susceptibility could occur prenatally. For example, it is plausible that the major window of susceptibility for lung is during in utero development, so sensitivity of the lung tissue would have been missed in this analysis (Miller 2004).

The acute studies exposures are largely by subcutaneous or intraperitoneal injection, which historically have not been considered relevant routes of environmental exposure for human cancer risk assessment by the U.S. EPA. For purposes of comparing age-dependent susceptibilities with tumor development, these data are highly relevant. The injection route typically alters the pharmacokinetic time courses of the parent compound and the metabolites compared with oral or other exposures because of altered kinetics of absorption and metabolism. However, for these compounds and the systemic organ effects observed, there are several pharmacokinetic reasons to believe that the age-dependent trends would be similar with other routes of exposure. These compounds are expected to be reasonably well absorbed orally, comparable with injection routes, and largely require metabolic activation, so partial or complete absence of first-pass metabolism in the injection studies would be similar to or underestimate metabolic activation compared with oral exposure. These studies provide the clearest demonstrations of periods of differential susceptibility because the exposure rate is constant at the different ages.

The information on life-stage susceptibility for chemicals inducing cancers through other than mutagenic modes of action is more varied, showing an increase in potency from perinatal exposure (e.g., PBBs induced liver tumors in female rats), no effect of combined perinatal and adult exposure (e.g., DPH liver tumors in rats), and different tumors from perinatal exposure versus adult exposure (e.g., DES, ascorbate). These variations are likely a result of the modes of action of these chemicals and the pharmacokinetic differences in doses during different periods of life.

An important factor that complicates the interpretation of the results for other modes of action is that these studies, except those with DDT and dieldrin, involved dietary feeding initially to the mother, which potentially could increase or decrease the dose received by the pups. Because of the maternal dosing during pregnancy and lactation, the extent to which offspring received similar doses during different early and adult life stages is particularly uncertain for DPH, ETU, and PBBs. Thus, these studies provide suggestive evidence that early life stages can be more sensitive to exposures to chemical causing cancer through a variety of modes of action other than mutagenicity. However, the studies with ETU indicate that this is not necessarily the case for all modes of action. No single-dose studies for chemicals with a nonmutagenic mode of action were evaluated that were directly comparable with the single-dose studies with mutagens.

There are important demonstrations of chemicals causing different tumor types with early–life-stage exposures compared with those for adults, for example, tamoxifen and DES (Carthew et al. 1996, 2000; Gass et al. 1964; Newbold et al. 1990, 1997). In addition, studies with in utero exposure to atrazine (Fenton and Davis 2002), DES, arsenic (Waalkes et al. 2003), and genistein (Newbold et al. 2001) indicate that early-life exposures to compounds can alter susceptibility of endocrine and reproductive organs. There is an actively growing database from which to consider issues of childhood exposure and cancer for compounds acting through the estrogen receptor or other mechanisms of endocrine disruption.

The ability to estimate with any accuracy the juvenile to adult cancer potency ratio depends on the experimental design used. The lifetime design has less ability to distinguish increased susceptibility from early-life exposure than the other study designs, as is more thoroughly explained with an example in the Appendix (http://ehp.niehs.nih.gov/docs/2005/7667/app.pdf).

The proper measure of relative potency of an exposure in the juvenile period relative to an exposure in the adult period is the ratio of doses in the two periods that give the same incidence of tumors. However, most of the data sets used in this report contained only one non-control dose, precluding the extensive dose–response modeling that would be required to estimate this ratio of doses. However, this analysis largely considered chemicals for which a mutagenic mode of action has been established and for which a linear, no-threshold dose–response function is assumed for the low-dose range being considered for risk assessment, and comparing potencies can be shown to be the same as comparing doses. This is illustrated in the Appendix (http://ehp.niehs.nih.gov/docs/2005/7667/app.pdf).

## Conclusions

In summary, the existing animal database supports the conclusion that there can be greater susceptibility for the development of tumors as a result of exposures early in life to chemicals acting through a mutagenic mode of action. Thus, a risk assessment approach using estimates from chronic studies with appropriate modifications to address the impact of early–life-stage exposure appears feasible. The U.S. EPA has recently released guidelines for multiplying an extra factor to the cancer potency for chemicals with a mutagenic mode of action for exposures that occur during childhood. The proposed factors are 10 for exposures to children between 0 and 2 years of age, and 3 for exposures to children between 2 and 15 years of age. The factor of 10 is based on the data derived from this analysis, and the factor of 3 represents a decline in potency expected to occur as children mature (U.S. EPA 2005). For chemicals acting through a nonmutagenic mode of action, the available data suggest that a range of approaches needs to be developed over time for addressing cancer risk estimates from childhood exposures. Development of such approaches requires additional research to provide an expanded scientific basis for their support, including additional research and the possible development of new toxicity testing protocols that consider early–life-stage dosing.

## Figures and Tables

**Table 1:** List of chemicals considered in the quantitative analysis for which there are both early-life and adult exposure reported in the same animal experiment.

Chemical	References	Study type	Mutagenic mode of action
Amitrole	Vesselinovitch 1983	Repeat dosing	
Benzidine	Vesselinovitch et al. 1975b	Repeat dosing	X
Benzo[*a*]pyrene	Vesselinovitch et al. 1975a	Acute exposure	X
Dibenzanthracene	Law 1940	Acute exposure	X
Dichlorodiphenyltrichloroethane	Vesselinovitch et al. 1979a	Repeat dosing Lifetime exposure	
Dieldrin	Vesselinovitch et al. 1979a	Repeat dosing Lifetime exposure	
Diethylnitrosamine	Peto et al. 1984	Lifetime exposure	X
	Vesselinovitch et al. 1984	Acute exposure	
Dimethylbenz[*a*]anthracene	Meranze et al. 1969	Acute exposure	X
	Pietra et al. 1961	Acute exposure	
	Walters 1966	Acute exposure	
Dimethylnitrosamine	Hard 1979	Acute exposure	X
Diphenylhydantoin, 5,5-	Chhabra et al. 1993b	Repeat dosing Lifetime exposure	
Ethylnitrosourea	Naito et al. 1981	Acute exposure	X
	Vesselinovitch et al. 1974	Acute exposure	
	Vesselinovitch 1983	Acute exposure	
Ethylene thiourea	Chhabra et al. 1992	Repeat dosing Lifetime exposure	
3-Methylcholanthrene^a^	Klein 1959	Repeat dosing	X
N-Methylnitrosourea	Terracini and Testa 1970	Acute exposure	
	Terracini et al. 1976	Acute exposure	X
Polybrominated biphenyls	Chhabra et al. 1993a	Repeat dosing Lifetime exposure	
Safrole	Vesselinovitch et al. 1979a	Repeat dosing Lifetime exposure	X
Urethane	Chieco-Bianchi et al. 1963	Acute exposure	X
	Choudari Kommineni et al. 1970	Acute exposure	
	De Benedictis et al. 1962	Acute exposure	
	Fiore-Donati et al. 1962	Acute exposure	
	Klein 1966	Acute exposure Lifetime exposure	
	Liebelt et al. 1964	Acute exposure	
	Rogers 1951	Acute exposure	
Vinyl chloride	Maltoni et al. 1984	Repeat dosing	X

X, chemicals with a mutagenic mode of action. The chemicals listed here are from the list of more than 50 chemicals found to have carcinogenic effects from prenatal or postnatal exposures in animals [Supplementary Table S1 (http://ehp.niehs.nih.gov/docs/2005/7667/supp.pdf)].

a Formerly known as 20-methylcholanthrene.

**Table 2:** Ratio of early-life to adult cancer potencies for studies with repeat exposures of juvenile and adult animals to mutagenic chemicals.

					Ratio of juvenile to adult cancer potency				
Compound	Species, strain	Sex	Dose	Tumor	Geometric mean	2.5%	Median	97.5%	Reference
Benzidine	Mice (B6C3F_1_)	Male		Liver	111	64	110	198	Vesselinovitch et al. 1975b
		Female		Liver	0.16	0.004	0.22	1.1	
3-Methylcholanthrene^a^	Mice (albino)	Male	0.25 mg/g	Hepatoma	33	7.4	30	268	Klein 1959
		Female	0.25 mg/g	Hepatoma	7.7	1.1	7.1	85	
		Male	0.25 mg/g	Forestomach	0.91	0.39	0.91	2.1	
		Female	0.25 mg/g	Forestomach	1.5	0.58	1.5	4.2	
		Male	0.25 mg/g	Skin	1.8	0.048	2.1	22	
		Female	0.25 mg/g	Skin	1.5	0.023	1.8	21	
Safrole	Mice (B6C3F_1_)	Male		Liver	46	16	44	198	Vesselinovitch et al. 1979b
		Female		Liver	0.12	0.002	0.18	1.1	
Vinyl chloride^b^	Rats (Sprague-Dawley)	Male	10,000 ppm	Liver angiosarcoma	7.4	0.035	11	62	Maltoni et al. 1984
		Female	10,000 ppm	Liver angiosarcoma	30	8.7	29	121	
		Male	10,000 ppm	Zymbal gland	0.27	0.0022	0.4	5.4	
		Female	10,000 ppm	Zymbal gland	0.15	0.0014	0.19	4.5	
		Male	10,000 ppm	Leukemia	21	0.026	37	514	
		Female	10,000 ppm	Leukemia	0.29	0.0019	0.35	17	
		Male	10,000 ppm	Nephroblastomas	0.17	0.0015	0.21	6.2	
		Female	10,000 ppm	Nephroblastomas	0.24	0.0017	0.29	11	
		Male	10,000 ppm	Angiosarcomas other sites	0.25	0.0017	0.30	12	
		Female	10,000 ppm	Angiosarcomas other sites	0.32	0.0019	0.38	20	
		Male	10,000 ppm	Angiomas and fibromas other sites	1.4	0.0045	2.36	47	
		Female	10,000 ppm	Angiomas and fibromas other sites	0.52	0.0024	0.63	41	
		Male	10,000 ppm	Hepatoma	34	8.2	32	218	
		Female	10,000 ppm	Hepatoma	55	8.4	53	513	
		Male	10,000 ppm	Skin carcinomas	0.41	0.0024	0.56	15	
		Female	10,000 ppm	Skin carcinomas	0.31	0.0019	0.37	19	
		Male	10,000 ppm	Neuroblastoma	0.20	0.0016	0.24	8.5	
		Female	10,000 ppm	Neuroblastoma	0.14	0.0014	0.18	4.4	

a Formerly known as 20-methylcholanthrene.

b Results for 6,000 ppm are similar to those for 10,000 ppm and are given in Supplementary Table S4 (http://ehp.niehs.nih.gov/docs/2005/7667/supp.pdf).

**Table 3:** Ratio of early-life to adult cancer potencies for studies with lifetime exposures starting with juvenile and adult, for chemicals acting through a mutagenic mode of action.

					Ratio of juvenile to adult cancer potency				
Compound	Species, strain	Sex	Dose	Tumor	Geometric mean	2.5%	Median	97.5%	References
Diethylnitrosamine	Rats (Colworth)		Multiple	Liver	2.8	0.0093	5.6	23	Peto et al. 1984
				Esophagus	0.18	0.0015	0.23	4.8	
Safrole	Mice (B6C3F_1_)	Male		Liver	46	3.7	50	253	Vesselinovitch et al. 1979b
		Female		Liver	1.9	0.007	4.0	23	
Urethane	Mice (B6AF_1_/J)	Male	2.5 μg/g/bw	Liver	79	0.36	102	1,064	Klein 1966
		Female	2.5 μg/g/bw	Liver	0.47	0.0022	0.55	43	

bw, body weight.

**Table 4:** Summary of quantitative estimates of ratio of early-life to adult cancer potencies.

Dose	Tissue	No. of chemicals	Geometric mean ratio	Range of ratios	No. of ratios
Chemicals with mutagenic mode of action					
Repeated		4	10.5	0.12–111	45
Lifetime		3	8.7	0.18–79	6
Combined repeated and lifetime		6	10.4	0.12–111	51
Acute					
	Combined (all tissues)	8	1.5	0.01–178	268
	Forestomach	3	0.076	0.01–1.9	32
	Harderian	2	0.48	0.06–0.8	20
	Kidney	2	1.6	0.17–7.1	18
	Leukemia	1	5.9	5.1–6.7	2
	Liver	5	8.1	0.10–40	70
	Lung	7	1.1	0.04–178	77
	Lymph	2	1.8	1.1–2.7	3
	Mammary					
	Week 5 vs. week 26	1	7.1	NA	1
	Week 2 vs. weeks 5–8 or 26	1	0.071	NA	2
	Nerve	2	2.3	0.24–64	10
	Nerve (day 1 comparison)	2	10	0.24–64	3
	Ovarian	1	0.033	0.01–0.13	3
	Reticular tissue	1	6.5	2.0–8.6	2
	Thymic lymphoma	1	2.8	1.0–7.9	6
	Thyroid	1	0.05	0.03–0.08	2
	Uterine/vaginal	1	1.6	0.03–8.6	3
	Day 1 (all tissues)	7	1.7	0.01–178	127
	Day 15 (all tissues)	3	1.5	0.06–52	74
Chemicals with nonmutagenic mode of action					
Repeated		6	2.2	0.06–13	22
Lifetime		5	3.4	0.15–36	38

NA, not applicable.

**Table 5:** Ratio of early-life to adult cancer potencies for studies with repeated exposures of juvenile and adult animals to nonmutagenic chemicals.

					Ratio of juvenile to adult cancer potency				
Compound	Species, strain	Sex	Dose	Tumor	Geometric mean	2.5%	Median	97.5%	References
Amitrole	Mice (B6C3F_1_)	Male	500	Liver	13	5.1	14	30	Vesselinovitch 1983
		Female	500	Liver	0.14	0.0013	0.18	3.9	
DDT	Mice (B6C3F_1_)	Male	150	Liver	1.3	0.0044	2.5	25	Vesselinovitch et al. 1979a
Dieldrin	Mice (B6C3F_1_)	Male	10	Liver	0.75	0.0031	1.2	27	Vesselinovitch et al. 1979a
DPH	Rats (F344/N)	Male	630	Liver	0.40	0.0024	0.54	16	Chhabra et al. 1993b
		Female	630	Liver	0.24	0.0017	0.29	12	
	Mice (B6C3F_1_)	Male	210	Liver	1.5	0.0040	2.4	71	
		Female	210	Liver	1.3	0.0056	2.6	15	
ETU	Rats (F344/N)	Male	90	Thyroid	0.37	0.0029	0.61	5.4	Chhabra et al. 1992
		Female	90	Thyroid	0.23	0.0018	0.30	7.0	
	Mice (B6C3F_1_)	Male	330	Liver	0.091	0.0011	0.12	1.9	
		Female	330	Liver	0.057	0.0010	0.081	0.65	
		Male	330	Thyroid	0.41	0.0022	0.52	25	
		Female	330	Thyroid	0.40	0.0024	0.55	16	
		Male	330	Pituitary	0.32	0.0019	0.38	22	
		Female	330	Pituitary	0.24	0.0018	0.32	6.9	
PBB	Rats (F344/N)	Male	10	Liver	0.59	0.0041	1.1	6.6	Chhabra et al. 1993a
		Female	10	Liver	0.063	0.0009	0.079	1.2	
		Male	10	Mononuclear cell leukemia	0.79	0.0035	1.4	18	
		Female	10	Mononuclear cell leukemia	0.21	0.0017	0.28	6.0	
	Mice (B6C3F_1_)	Male	30	Liver	3.9	1.9	3.9	7.5	
		Female	30	Liver	1.0	0.37	1.05	2.1	
